# Exploring the Association of *FTO* rs9939609 with Type 2 Diabetes, Fasting Glucose and HbA1c in a Southeastern Mexican Region of Predominant Mayan Genetic Background

**DOI:** 10.3390/biom15111492

**Published:** 2025-10-23

**Authors:** Nicolas Fragoso-Bargas, Litzy Naomi Toloza-Couoh, Irma Quintal-Ortiz, Guillermo Valencia-Pacheco, Nina Valadez-Gonzalez

**Affiliations:** 1 Mohn Center for Diabetes Precision Medicine, Department of Clinical Science, University of Bergen, 5009 Bergen, Norway; 2 Hematology Laboratory, Regional Research Center “Dr. Hideyo Noguchi”, Autonomous University of Yucatán, Yucatán 97225, Mexico; naomitoloza@gmail.com (L.N.T.-C.); qortiz@correo.uady.mx (I.Q.-O.); vpacheco@correo.uady.mx (G.V.-P.); valadez@correo.uady.mx (N.V.-G.)

**Keywords:** Type 2 diabetes, FTO, rs9939609, SNP, SNV, genetic variant, polymorphism, Maya, biomarkers, glucose, HbA1c

## Abstract

Type 2 diabetes (T2D) is a multifactorial disease characterized by chronic hyperglycemia. The *FTO* variant rs9939609 has been widely associated with obesity, but emerging evidence suggests a broader role for T2D risk across diverse populations. However, Mayan ancestry individuals remain underrepresented in genetic studies. Thus, we evaluated the association of rs9939609 with T2D, fasting glucose, and HbA1c in a southeastern Mexican region with prevalent Mayan ancestry. Birthplace was used as a proxy for ancestry, although no formal ancestry assessment was conducted. The A allele was associated with increased risk for T2D in both additive (OR = 1.88 [1.08–3.40], *p* = 0.031) and dominant (OR = 2.09 [1.08–4.15], *p* = 0.032) models, even after adjusting for age, sex, BMI, waist circumference, and waist/hip ratio. The A allele was also associated with increased fasting glucose and HbA1c levels in the dominant model. Mediation analysis suggested that T2D may mediate the effect of rs9939609-A on glucose traits. rs9939609-A may be a risk factor for T2D and elevated glucose levels in this population. However, the results should be interpreted with caution due to the limited sample size, which may result in under- or overestimation of effect sizes.

## 1. Introduction

Type 2 diabetes (T2D) is a multifactorial disease characterized by the progressive dysfunction of beta-cells often accompanied by insulin resistance [1]. When beta-cells can no longer compensate for the increased insulin demand, disturbances in glucose homeostasis arise, ultimately leading to chronic hyperglycemia, a key criterion for the clinical diagnosis of T2D. Hyperglycemia is typically assessed via fasting glucose, oral glucose tolerance, or HbA1c levels [2]. In Mexico, the prevalence of T2D among individuals over 20 years of age is estimated at 18.3% [3], making it the third leading cause of death nationwide [4]. Several risk factors have been associated with T2D risk, such as obesity, family history, physical inactivity, age, and ancestry, particularly among Hispanics/Latinos and Native Americans [5].

The biggest GWAS meta-analysis has successfully identified ~1300 genetic variants associated with T2D risk [6]. While Hispanic individuals are represented in this study, the majority of participants are of European ancestry. Moreover, the Hispanic cohort from Mexico [7] mainly included admixed individuals from Mexico City, whose Amerindian ancestral background comprises mainly of Indigenous groups such as the Nahuatl, Mazahua and Otomi [8]. On the other hand, in the southeastern region of Mexico, specifically on the Yucatan Peninsula, Mayan ancestry is predominant [9]. Compared to other Mexican pre-Hispanic ancestries, individuals with Maya ancestry have a higher prevalence of T2D [9]. However, southeastern Mexican populations from the Yucatan peninsula, where Mayan ancestry is prevalent, remain underrepresented in genetic association studies.

Among the efforts to identify T2D-associated variants in populations in southeastern Mexico, a GWAS identified the rs1914711-G variant near *AGTR2* [10], although the sample size was limited (*n* = 92), reducing the power for locus discovery. Similarly, an exome sequencing study (*n* = 24) found that rs1799999-A near *PPP1R3A* was associated with an increased risk of T2D [11]. Candidate gene studies have also reported associations between higher T2D risk and variants such as rs10811661-T (*CDKN2A/2B*) [12], rs9282541-T (*ABCA1*) [12], rs1799987-A (*CCR5*) [13], rs7896005-G (*SIRT1*) [14], and rs6446482-G (*WFS1*) [14], while rs1800795-C (*IL6*) has been suggested as a protective factor in males [15]. Given the limited number of SNPs currently identified as associated with T2D risk, further research is needed to identify the genetic factors contributing to T2D in the southeastern region of Mexico.

The fat mass and obesity (*FTO*) is a gene over 400kb long located in the long arm of chromosome 16 (16a12.2) that contains 9 exons and 8 introns [16]. *FTO* encodes a deacetylase involved in several metabolic processes and traits, including adipocyte differentiation, appetite regulation, lipid accumulation, satiety, and body mass index (BMI) [16]. *FTO* harbors the genetic variant rs9939609-A, which has been primarily associated with higher BMI and obesity [17,18,19,20]. However, several studies across different populations have shown that rs9939609-A is also associated with an increased risk of T2D [21,22,23].

In Central and Northern Mexican populations, the rs9939609-A allele has been associated with an increased risk of metabolic syndrome [24], as well as elevated serum insulin levels [25]. A study in Mayan children also examined its association with fasting glucose, insulin, and HOMA-IR, though no statistically significant findings were reported [26]. Additionally, evidence from Chiapas, a region with predominant Mayan ancestry, suggests a possible link between rs9939609-A and hyperglycemia, categorized as fasting glucose ≥100 mg/dL [27]. To date, no study has reported the association between rs9939609-A and diagnosed T2D in adult individuals from Southeastern Mexico. Therefore, the aims of this study were to: (1) evaluate if rs9939609-A is associated with T2D and (2) assess whether rs9939609-A is associated with fasting glucose and HbA1c levels in a southeastern region (Yucatan, México) where Mayan ancestry is predominant.

## 2. Materials and Methods

### 2.1. Study Design and Participants

The study population consisted of residents from the locality of San José Tecoh, Mérida, Yucatán, Mexico. All participants were recruited at the health center “Unidad Universitaria de Inserción Social (UUIS) San José Tecoh” between 2018 and 2019. For cases, the inclusion criteria were: (1) diagnosis of T2D according to the Mexican diagnostic criteria NOM-015-SSA2-2010, which include a fasting plasma glucose ≥126 mg/dL, a random plasma glucose ≥200 mg/dL, or a plasma glucose ≥200 mg/dL two hours after an oral load of 75 g of anhydrous glucose dissolved in water; (2) individuals of any sex; (3) age over 20 years; and (4) being born in Mérida, Yucatán. For controls, the inclusion criteria were: (1) no family history of T2D in at least two generations; (2) individuals of any sex; (3) age over 20 years; and (4) being born in Mérida, Yucatán. Among the general exclusion criteria were: having a relative already included in the study, being pregnant, and having a diagnosis of type 1 diabetes (for case participants). Although ancestry markers were not measured in all individuals, a previously analyzed subset of controls (*n* = 37) included in this study showed evidence of Mayan ancestry [10,28].

All participants signed an informed consent form, indicating their voluntary participation, in accordance with the recommendations of the Declaration of Helsinki and the General Health Law. The study was approved by the Ethics Committee of the Dr. Hideyo Noguchi Regional Research Center at the Autonomous University of Yucatán (UADY).

### 2.2. Anthropometrics and Biochemical Measurements

Age, height, weight, waist circumference and hip measurements were obtained at recruitment, and BMI and waist-hip-ratio were subsequently calculated. Fasting plasma glucose and whole blood HbA1c were measured in both cases and controls using the same Roche Cobas^®^ c111 analyzer (Roche Diagnostics Ltd., Rotkreuz, Switzerland), following the manufacturer’s instructions. All individuals included in this study have complete data for these variables.

### 2.3. Genotyping

DNA was extracted using the Macherey-Nagel^®^ kit (Macherey-Nagel GmbH & Co. KG, Düren, Germany) from 200 µL of peripheral blood, following the manufacturer’s instructions. Rs9939609 genotyping was performed using allele discrimination assays with the specific TaqMan^®^ probe (Applied Biosystems, Foster City, CA, USA) C__30090620_10, following the manufacturer’s instructions. The amplification program began with an initial activation at 95 °C for 10 min, followed by 40 amplification cycles: each cycle included a denaturation step at 92 °C for 15 s and an annealing/extension step at 60 °C for 90 s. Each assay included both positive and negative controls.

### 2.4. Statistical Analyses

Hardy–Weinberg equilibrium was evaluated in cases, controls and all the study population using the R package “HardyWeinberg” (version 1.7.9) [29,30,31]. The Exact test for Hardy–Weinberg equilibrium described by Wigginton and collaborators [32] was used since it adequately controls for type I error in both large and small datasets.

For the association analyses, we used the additive (TT = 0, AT = 1, AA = 2), dominant (TT = 0, AT/AA = 1), and recessive (TT/AT = 0, AA = 1) models. The association between T2D and rs9939609 was evaluated using both univariable and multivariable logistic regression models. The multivariable models were adjusted for age and sex since both are well-established demographic factors that influence metabolic traits and disease risk. Additionally, given the strong association between *FTO* and adiposity, we adjusted for BMI, waist circumference, and waist/hip ratio. However, since these three variables are all proxies for adiposity and are highly correlated, we constructed separate models for each rather than including them simultaneously to avoid redundancy. For fasting glucose and HbA1c, we used three types of linear models: (1) univariable (i.e., unadjusted); (2) adjusted for age, sex, and one adiposity-related measure; and (3) the same as the previous model, with additional adjustment for T2D status. All logistic and linear models were constructed in R Studio (version 2025.09.1+401).

If a significant association was found between a glucose-related quantitative trait and rs9939609, we conducted a mediation analysis using the R package mediation (version 4.5.1) [33] to explore whether the association was mediated by T2D status. The mediation analysis estimates the following effects: (1) the Average Causal Mediation Effect (ACME), which quantifies the indirect effect, i.e., the portion of the association transmitted through the mediator (T2D); (2) the Average Direct Effect (ADE), which captures the direct effect of rs9939609 on the trait, adjusting for the mediator; (3) the Total Effect, which is the sum of the ACME and ADE; and (4) the Proportion Mediated, which represents the fraction of the total effect explained by the mediation pathway. To estimate the effects and their confidence intervals, we used the percentile method with 1000 simulations, setting a random seed of 100 to ensure reproducibility.

For the association and mediation analyses, we considered a *p*-value <0.05 as significant and a *p*-value <0.1 as a statistical trend.

## 3. Results

### 3.1. Population Characteristics

Clinical characteristics of the study population (*n* = 184) can be consulted in Table 1. Variables include age, sex distribution, fasting glucose, HbA1c, BMI, waist circumference and waist/hip ratio. There were 92 individuals with T2D (cases) and 92 without (controls), and 72% were females. There was no missing data.

### 3.2. Frequency Distribution and Associations with T2D

Genotype and allele frequencies can be consulted in Table 2. The minor allele frequency of the risk allele (A) was 15.2% for the entire population, 19.6% in cases and 10.9% in controls. We did not observe significant deviation from Hardy–Weinberg equilibrium in cases (*p* = 0.323), controls (*p* = 0.276), or the overall sample (*p* = 0.146). According to the univariable logistic regression model, rs9939609 conferred a 1.88-fold increased risk of T2D per copy of the A allele under the additive model (Table 3) and a 2.09-fold increased risk under the dominant model. The association with T2D remained significant in both the additive and dominant models, even after adjusting for covariates (Table 2). No significant association was identified in the recessive model.

### 3.3. Associations with Fasting Glucose and HbA1c

The univariable linear regression model showed that the A allele of rs9939609 was associated with increased fasting glucose levels under both the additive and dominant models, while for HbA1c, the association was observed only under the dominant model (Table 3). For both traits, associations under the dominant model remained significant after adjusting for age, sex, and an adiposity measurement, but were no longer significant in models further adjusted for T2D status (Table 3).

### 3.4. Mediation Analysis

**Figure 1:**
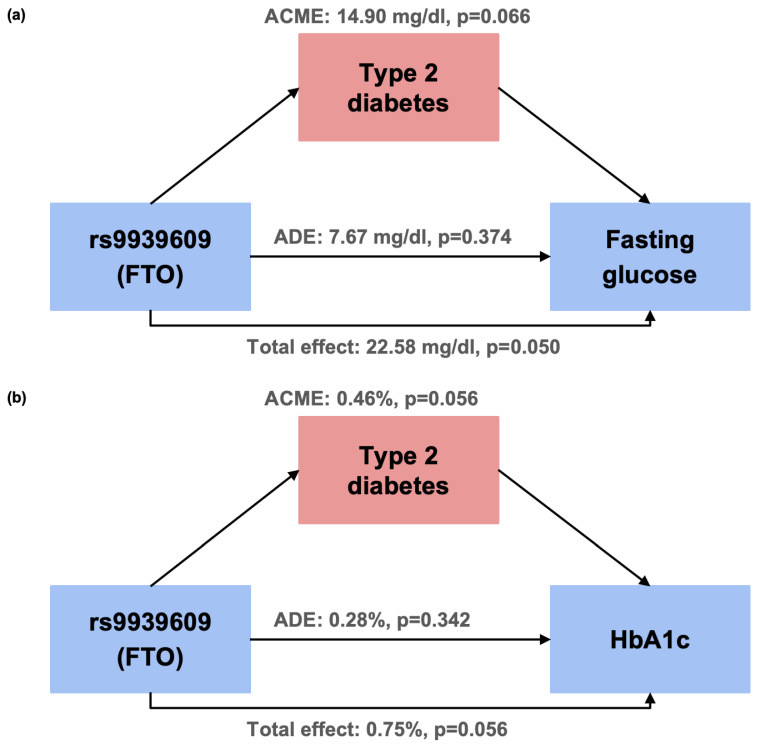
Schematic representation of the potential mediation pathway between rs9939609 and fasting glucose (**a**), and HbA1c (**b**), with type 2 diabetes as a potential mediator. ACME: Average Causal Mediation Effect; ADE: Average Direct Effect.

Since we observed consistent significant associations between rs9939609-A and both fasting glucose and HbA1c levels under the dominant model, which disappeared after adjusting for T2D, we performed mediation analyses based on the dominant model to explore whether T2D mediates this effect. The results provide suggestive evidence (*p* < 0.1) that T2D may mediate the relationship between rs9939609-A and both glucose traits, given that the ACME was stronger than the ADE (Table 4 and Figure 1). Additionally, the analysis suggests (*p* < 0.1) that T2D may explain up to 66% of this relationship (Table 4) for fasting glucose and 62% for HbA1c; however, the confidence intervals were wide.

## 4. Discussion

In this study, we provide evidence that rs9939609-A may be a genetic risk factor for T2D in a southeastern Mexican population. This association remains after adjusting for adiposity traits under both the additive and dominant models. Furthermore, we also observed that rs9939609-A is associated with increased glucose and HbA1c levels mainly under the dominant model. Mediation analysis provided suggestive evidence that T2D may be a mediator between rs9939609 and fasting glucose levels.

In our population, the A allele conferred an increased risk for T2D in both univariable and multivariable logistic regression under both additive and dominant genetic models. This is in line with a multi-ancestry meta-analysis, which concluded that rs9939609-A is consistently associated with T2D risk [21]. One proposed biological mechanism by which intronic *FTO* SNPs may increase T2D risk is through their influence on the expression of adjacent genes, *IRX3* and *IRX5*, which modulate brown adipocyte differentiation [34,35], as well as through appetite regulation via the hypothalamus [36,37]. Thus, rs9939609-A and other *FTO* polymorphisms may contribute to T2D risk by dysregulating adipocyte differentiation and appetite control. However, our results suggest that the association is independent of adiposity, as it persisted after adjusting for BMI, waist circumference, and waist/hip ratio. Consistent with this, previous studies in Norwegian [22] and Vietnamese populations [38] have also shown that the association with T2D remains significant after adjusting for BMI. This suggests that rs9939609 could act through pathways not solely related to adiposity, which remain to be elucidated. However, further verification in other Mayan or other indigenous populations are needed to verify the robustness of this relationship.

Regarding the positive association of rs9939609-A with both fasting glucose and HbA1c observed mainly under the dominant model, other populations have reported associations with glucose measurements. The A allele has been associated with increased glucose levels in Middle Eastern populations [39] and with both fasting glucose [40] and HbA1c [41] in South Asian populations. Moreover, a population from Chiapas, Mexico, who also has predominance of Mayan ancestry, suggested that rs9939609-A is associated with hyperglycemia, categorized as fasting glucose levels ≥ 126 mg/dL [27]. Taken together, these findings support the relevance of rs9939609-A in glycemic homeostasis regulation within populations with predominantly Mayan descent and highlight the transferability of rs9939609-A as a risk factor for both T2D and elevated glucose levels across diverse populations worldwide.

Our analyses suggest that the associations between rs9939609 and both fasting glucose and HbA1c may be influenced by T2D status. To explore this, we conducted a mediation analysis to evaluate whether T2D acts as a potential mediator between rs9939609 and these glycemic traits. The ACME model indicated that T2D may mediate the effect of rs9939609 on both outcomes. This suggests that the variant could influence fasting glucose and HbA1c levels indirectly by increasing the risk of T2D, rather than exerting a direct effect in non-diabetic individuals. While this finding is intriguing, it should be interpreted with caution. The significance of the ACME model was only a statistical trend (*p* > 0.05 and <0.1). Furthermore, the mediation analysis was not intended to establish causality but rather to serve as an exploratory, hypothesis-generating approach, given our limited sample size. It is also important to note that our study design includes a balanced number of T2D cases and controls, which may influence these observations. Larger studies in Mayan or other populations are needed to confirm this potential pathway.

An important limitation of this study is that ancestry markers have been not assessed in all the study population. Nevertheless, birthplace in Mérida was used as a proxy for regional ancestry, given that Mayan ancestry is the predominant Indigenous component in the Yucatán Peninsula [9]. Furthermore, a previous study that included 45 individuals from the municipality of Mérida, 37 of whom are also part of this study, compared their ancestry proportions with those of 47 individuals from another municipality in Yucatán (Sisal) [10,28]. Both populations, despite being from distinct localities, showed evidence of Mayan ancestry. Hence, although the ancestry markers were not assessed on all individuals, it is highly likely that most participants in this study have Mayan ancestry. Moreover, the same study identified a statistical trend between rs9939609-A and T2D, with a coefficient consistent with our direction of effect (OR = 1.32, *p* = 0.080). This suggests that the significant association observed in our study may be driven by the larger sample size, and that the lack of ancestry confirmation in all individuals likely does not affect the interpretation of this association, given the consistent effect direction observed in a previous analysis of confirmed Mayan individuals. Finally, the replication of the association between rs9939609-A and T2D in our cohort, along with previous evidence linking this variant to hyperglycemia in another region with a high prevalence of Mayan ancestry, supports the robustness of our findings.

Strengths of this study include a balanced number of cases and controls, which enhances statistical power and reduces bias. Carefully selected controls with no family history of diabetes for at least two generations, strengthening the validity of the control group, and the use of quantitative glucose measurements, allowing for the assessment of genetic associations beyond binary T2D status and mediation analysis. However, some limitations should be acknowledged. Since the sample size was limited, the presented associations should ideally be replicated in a larger study. Therefore, our results should be interpreted with caution because it is likely that our effect sizes reported are either under- or overestimated. Additionally, the lack of ancestry-informative marker measurements in all subjects limits our ability to precisely verify the presence and proportion of Mayan ancestry among participants.

## 5. Conclusions

To conclude, our findings support the role of rs9939609-A as a genetic risk factor for T2D in a population from southeastern México, a region where Mayan ancestry is predominant. This association is robust after adjusting for adiposity-related measurements. These results contribute to the growing evidence of *FTO*’s pleiotropic effects, as it may influence glycemic traits through mechanisms beyond adiposity. Future studies in similar populations using ancestry-informative markers or with increased statistical power are needed to validate and extend the presented findings.

## Figures and Tables

**Table 1:** Clinical characteristics of the study population. Data are mean (SD) for quantitative measures and *n* (%) for categorical variables).

Phenotype	All (*n* = 184)	Cases (*n* = 92)	Controls (*n* = 92)
Age	58.21 (10.78)	56.83 (10.20)	59.60 (11.21)
Sex (female)	133 (72)	70 (76)	63 (69)
Fasting glucose (mg/dL)	121.52 (53.88)	155.50 (57.36)	87.53 (14.59)
HbA1c (%)	6.69 (2.02)	8.08 (1.94)	5.29 (0.70)
BMI (kg/m^2^)	29.60 (5.07)	30.30 (5.55)	28.89 (4.47)
Waist circumference (cm)	95.66 (10.81)	97.79 (10.97)	93.54 (10.28)
Waist/hip ratio	0.91 (0.06)	0.91 (0.06)	0.91 (0.06)

**Table 2:** Genotype and allele frequency distribution between cases and controls, and logistic regression analyses evaluating the association between rs9939609 and T2D risk. The model column indicates whether the analysis was unadjusted or adjusted for specific covariates. Data are *n* (%) for genotype and allele frequencies.

Genotype and Allele frequencies							
Genotype	All	Cases	Controls	Allele	All	Cases	Controls
AA	7 (3.8)	5 (5.4)	2 (2.2)	A	56 (15.2)	36 (19.6)	20 (10.9)
AT	42 (22.8)	26 (28.3)	16 (17.4)	T	312 (84.8)	148 (80.4)	164 (89.1)
TT	135 (73.4)	61 (66.3)	74 (80.4)				
Association analyses with T2D							
Model	Additive OR (IC)	*p*	Dominant OR (IC)	*p*	Recessive OR (IC)		*p*
Unadjusted	1.88 (1.08–3.40)	**0.031**	2.09 (1.08–4.15)	**0.032**	2.59 (0.54–18.39)		0.264
Age + Sex + BMI	1.89 (1.07–3.47)	**0.032**	2.14 (1.09–4.30)	**0.030**	2.43 (0.51–17.36)		0.297
Age + Sex + Waist	1.88 (1.06–3.47)	**0.036**	2.11 (1.06–4.29)	**0.036**	2.54 (0.52–18.46)		0.280
Age + Sex + WHR	1.93 (1.09–3.55)	**0.027**	2.18 (1.10–4.39)	**0.027**	2.58 (0.52–18.75)		0.274

Abbreviations: Waist: Waist circumference, WHR: waist/hip ratio. Highlighted in bold are *p* < 0.05.

**Table 3:** Linear regression analyses of the association between rs9939609 and fasting glucose and HbA1c. The model column indicates whether the analysis was unadjusted or adjusted for specific covariates.

Phenotype	Model	Additive	*p*	Dominant	*p*	Recessive	*p*
		Beta (SE)		Beta (SE)		Beta (SE)	
Glucose (mg/dL)	Unadjusted	14.54 (7.34)	**0.049**	19.76 (8.89)	**0.027**	8.85 (20.81)	0.671
	Age + Sex + BMI	7.26 (1.98)	**0.049**	19.88 (8.79)	**0.025**	6.94 (20.60)	0.737
	Age + Sex + BMI + T2D	4.61 (5.86)	0.432	8.11 (7.09)	0.254	−6.88 (16.28)	0.673
	Age + Sex + Waist	13.57 (7.09)	0.057	18.66 (8.59)	**0.031**	7.21 (20.08)	0.720
	Age + Sex + Waist + T2D	4.52 (5.80)	0.436	7.88 (7.03)	0.264	−6.34 (16.13)	0.694
	Age + Sex + WHR	14.61 (7.17)	**0.043**	20.15 (8.68)	**0.021**	7.43 (20.35)	0.716
	Age + Sex + WHR + T2D	4.85 (5.79)	0.403	8.38 (7.00)	0.233	−6.49 (16.09)	0.687
HbA1c (%)	Unadjusted	0.52 (0.28)	0.061	0.78 (0.33)	**0.020**	−0.08 (0.78)	0.914
	Age + Sex + BMI	0.52 (0.27)	0.058	0.79 (0.33)	**0.016**	−0.17 (0.77)	0.829
	Age + Sex + BMI + T2D	0.11 (0.20)	0.585	0.31 (0.25)	0.215	−0.74 (0.56)	0.186
	Age + Sex + Waist	0.49 (0.27)	0.067	0.76 (0.32)	**0.020**	−0.16 (0.76)	0.833
	Age + Sex + Waist + T2D	0.11 (0.20)	0.589	0.30 (0.24)	0.219	−0.74 (0.56)	0.190
	Age + Sex + WHR	0.52 (0.27)	0.053	0.80 (0.32)	**0.015**	−0.15 (0.76)	0.841
	Age + Sex + WHR + T2D	0.12 (0.20)	0.564	0.31(0.24)	0.202	−0.73 (0.56)	0.188

Abbreviations: Waist: Waist circumference, WHR: waist/hip ratio. Highlighted in bold are *p* < 0.05.

**Table 4:** Mediation analysis evaluating T2D as a mediator in the relationship between rs9939609 and fasting glucose and HbA1c.

Glucose (mg/dL)			
Model	Effect	CI 95%	*p*-Value
ACME (mg/dL)	14.90	−1.053, 25.00	0.066
ADE (mg/dL)	7.67	−8.56, 24.32	0.374
Total Effect (mg/dL)	22.58	0.01, 39.92	0.050
Proportion Mediated	66	−35, 242	0.082
**HbA1c (%)**			
ACME (%)	0.46	−0.02, 0.100	0.056
ADE (%)	0.28	−0.26, 0.85	0.342
Total Effect (%)	0.75	−0.01, 1.56	0.056
Proportion Mediated (%) *	62	−11, 226	0.060

ACME: Average causal mediation effect, ADE: Average direct effect. * Values represent mediation proportions expressed as percentages (0–100%), not HbA1c concentration percentages.

## Data Availability

To protect patient rights and ensure anonymity, the data are available upon reasonable request.

## Abbreviations

The following abbreviations are used in this manuscript:

ADE	Average Direct Effect
ACME	Average Causal Mediation Effect
BMI	Body mass index
FTO	Fat mass and obesity-associated
SNV	Single-nucleotide variant
T2D	Type 2 diabetes
